# Cone–Rod Dystrophy *PCARE*-Associated Retinopathy

**DOI:** 10.3390/diagnostics16131945

**Published:** 2026-06-23

**Authors:** Maria Sopena-Pinilla, Maria Arruebo-Muñio, Marta Arias-Alvarez, Maria Arcas-Carbonell, Pablo Tejada-González, Carmen Lahuerta-Pueyo, Diana Pérez García, Isabel Pinilla

**Affiliations:** 1 Department of Ophthalmology, Hospital Universitario Miguel Servet, 50009 Zaragoza, Spain; mariasopenapinilla@gmail.com; 2 Aragon Institute for Health Research (IIS Aragon), 50009 Zaragoza, Spain; marcas@unizar.es; 3 Department of Biochemistry, Hospital Clínico Universitario Lozano Blesa, 50009 Zaragoza, Spain; mam.arruebo@gmail.com (M.A.-M.); carmenlpueyo@gmail.com (C.L.-P.); 4 Department of Neurophysiology, Hospital Clínico Universitario Lozano Blesa, 50009 Zaragoza, Spain; martariasalvarez7@gmail.com; 5 Department of Ophthalmology, Hospital Clínico Universitario Lozano Blesa, 50009 Zaragoza, Spain; tejadagonzalezpablo@gmail.com (P.T.-G.); dianapgpe@hotmail.com (D.P.G.); 6 Department of Surgery, University of Zaragoza, 50009 Zaragoza, Spain

**Keywords:** *PCARE*, inherited retinal dystrophies, macular atrophy

## Abstract

**Background and Clinical Significance**: Biallelic pathogenic variants in the *PCARE* gene (*photoreceptor cilium actin regulator*), also known as *C2orf71* (*chromosome 2 open reading frame 71*), are typically associated with retinitis pigmentosa type 54 (RP54) and, less frequently, with cone–rod dystrophy (CORD23). **Case Presentation**: A 52-year-old man presented with an eight-year history of progressive visual loss, without photophobia or nyctalopia. He underwent a comprehensive ophthalmological evaluation, including multimodal retinal imaging, automated perimetry, and full electrophysiological testing, in accordance with International Society for Clinical Electrophysiology of Vision (ISCEV)’s standards. Genetic testing was performed using next-generation sequencing (NGS) with an inherited retinal dystrophy gene panel, and findings were confirmed by Sanger sequencing. Clinical examination revealed bilateral macular atrophy with minimal foveal sparing and a central scotoma. Optical coherence tomography (OCT) showed disruption of the outer retinal layers and retinal pigment epithelium (RPE) abnormalities. Fundus autofluorescence (FAF) demonstrated central hypoautofluorescence surrounded by a hyperautofluorescent ring. Electrophysiological testing revealed severely reduced rod- and cone- mediated responses on full-field electroretinography (ERG), absent pattern ERG responses, and markedly reduced multifocal ERG responses, indicating widespread retinal dysfunction with significant macular involvement. Genetic analysis identified a homozygous pathogenic nonsense variant in *PCARE* [c.3289C>T; p.(Gln1097*)], confirming the diagnosis of an autosomal recessive inherited retinal dystrophy. **Conclusions**: Biallelic *PCARE* variants can cause late-onset severe retinal dystrophy, with predominant macular involvement and cone–rod dysfunction. Given its phenotypic overlap with other inherited retinal diseases, accurate diagnosis requires the integration of multimodal retinal imaging, electrophysiological testing, and comprehensive genetic analysis.

## 1. Introduction

*PCARE* (*photoreceptor cilium actin regulator*), also known as *C2orf71* (*chromosome 2 open reading frame 71*), is a retina-specific gene predominantly localized in photoreceptors at the outer segment and/or the connecting cilium. Studies have shown that *PCARE* interacts with proteins involved in actin remodeling, such as WASF3, to regulate actin dynamics and promote ciliary membrane expansion, a process that is essential for the development and maintenance of photoreceptor outer segments. The encoded protein is also believed to undergo post-translational lipid modification [1].

*C2orf71* encodes a putative protein of 1289 amino acids that is specifically expressed in the human retina at the site of the outer segment disk formation [2]. It is thought to play a critical role in actin-dependent processes required for photoreceptor morphogenesis and maintenance. Because of its involvement in ciliary function, retinal dystrophies associated with *PCARE* variants are classified as retinal ciliopathies.

Pathogenic variants in *C2orf71* are primarily associated with autosomal recessive retinitis pigmentosa (RP), specifically RP54 (OMIM #613428) [3,4]. This condition presents with a classical RP phenotype, including night blindness, bone-spicule-type pigment deposits in the peripheral retina and attenuated retinal vessels. However, early cone photoreceptor degeneration is also common and may manifest as a ring scotoma on visual field testing [4]. Less commonly, *PCARE* variants have been implicated in cone–rod dystrophy (CORD23) [5,6]. Inherited retinal dystrophies (IRD) caused by *PCARE* mutations generally present during the second or third decade of life, with nyctalopia being a common early symptom. Fundus findings are variable, ranging from a typical RP appearance to early-onset maculopathy. The prevalence of *PCARE*-associated IRDs appears to vary across populations. Tiwari et al. reported that *PCARE* mutations represent the second-most common genetic cause of IRD in a Swiss cohort (15.38%; *n* = 58) [7], whereas in a French series of 209 patients, only 1% were associated with novel *PCARE* mutations [8]. Most pathogenic variants are single nucleotide changes that result in premature stop codons, resulting in loss of protein function.

Cone dystrophies (COD) and CORD are a group of IRDs characterized by primary cone degeneration, which may be followed by secondary rod involvement [9]. Clinically, these disorders present with progressive central visual loss, photophobia, and dyschromatopsia. Although variants in *ABCA4* represent the most common genetic cause of autosomal recessive CORD, *PCARE* is a rare but well-established causative gene. *PCARE*-associated CORD is typically characterized by central visual impairment and a parafoveal ring scotoma, which may progress to extensive macular chorioretinal atrophy [5].

Here, we report the case of a 52-year-old man who was initially diagnosed with type 1 Stargardt disease but was subsequently found to have cone–rod dystrophy caused by a homozygous pathogenic *PCARE* variant. This case highlights the phenotypic overlap among inherited retinal diseases and underscores the importance of comprehensive electrophysiological and genetic evaluation for establishing an accurate diagnosis.

## 2. Materials and Methods

A 52-year-old Caucasian male presented with an 8-year history of progressively declining distance visual acuity (VA), which had further deteriorated in near vision over the past two years. The patient did not report either photophobia or nyctalopia.

The patient’s family originates from Eastern Europe. His paternal lineage included four unaffected uncles, three aunts with normal vision, and one aunt who was blind. On his maternal side, there were 3 unaffected aunts and one uncle with impaired VA. Additionally, one of his two sisters reported visual problems. None of these relatives were available for clinical evaluation in Spain. The patient has two healthy sons with no visual symptoms, and he denied any family history of consanguinity.

### 2.1. Ophthalmic Evaluation

A comprehensive ophthalmological assessment was performed. Best corrected VA (BCVA) was measured using Early Treatment Diabetic Retinopathy Study’s (ETDRS) charts. Anterior segment examination was conducted by slit lamp biomicroscopy. Fundus examination was performed under pharmacological pupil dilation. Retinal imaging included widefield color fundus photography (Clarus 700, Carl Zeiss Meditec, Dublin, OH, USA), spectral-domain optical coherence tomography (OCT), and fundus autofluorescence (FAF) using the Spectralis OCT system (Heidelberg Engineering, Heidelberg, Germany). OCT angiography (OCTA) was performed using a swept-source OCT device (Triton, Topcon Corporation, Tokyo, Japan). Retinal function was evaluated by standard automated perimetry (Humphrey 24-2 visual field) and electrophysiological testing (RETI-port/scan21 recording software, Version 1021.3.0.0, released on 31 May 2021, Roland Consult, Brandenburg, Germany), conducted in accordance with the standards of the International Society for Clinical Electrophysiology of Vision (ISCEV) [10,11].

### 2.2. Genetic Study

Genomic DNA (gDNA) was extracted from peripheral blood leukocytes using the magLEAD 12gC system (Precision System Science, Chiba, Japan).

Next-generation sequencing (NGS) was performed using a retinal dystrophy gene panel comprising 322 genes associated with inherited retinal diseases (Appendix A). The analysis included all clinically relevant coding exons and selected intronic regions. Library preparation and enrichment were performed using the Twist Comprehensive Exome kit (Twist Bioscience, San Francisco, CA, USA), and sequencing was carried out on an Illumina NextSeq 1000 platform (San Diego, CA, USA). Bioinformatic analysis and variant interpretation were conducted using Sophia DDM software.

This CE-IVD-certified approach achieved near-complete coverage for the vast majority of targeted genes, with a minimum depth of 30× across nearly all analyzed regions. Complete coverage was not achieved for *ARV1*, *B3GALNT2*, *BMP4*, *CCDC28B*, *CNGA1, HK1*, *KIAA0586*, *NUDFB11*, *NEK2*, *REEP6*, *RPGR*, and *VPS13B*; however, the lowest coverage observed was 73.7% at 30× for *CCDC28B*. In addition, this method enables the detection of CNVs (Copy Number Variations) across all genes included in the panel.

The identified pathogenic variant was confirmed by Sanger sequencing using an ABI 3130xl Genetic Analyzer (Applied Biosystems Life Technologies, Foster City, CA, USA).

## 3. Results

### 3.1. Clinical Findings

**Figure 1:**
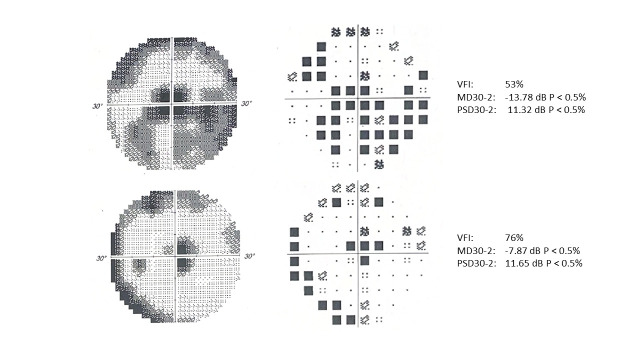
Humphrey field analyzer visual field testing; grayscale plots, pattern deviation plots, and sensitivity maps of the right eye (top row) and left eye (bottom row). Both eyes demonstrate a central scotoma and reduced retinal sensitivity extending into the mid-peripheral visual field. Test reliability was limited by a high rate of false-negative responses.

BCVA was 20/32 in both eyes. The spherical equivalent (SE) was +0.75 diopters in the right eye (RE) and +1.25 diopters in the left eye (LE). Slit lamp examination revealed mild posterior subcapsular lens opacification bilaterally. Intraocular pressure (IOP) was 16 mmHg in both eyes. Visual field testing showed a high false negative rate in both eyes and abnormal results, with central scotomas and mild peripheral sensitivity loss (MD 30-2 −13.78 dB for RE and −7.98 dB for LE) (Figure 1). Microperimetry examination (MAIA, Microperimeter Macular Integrity Assessment; CenterVue SpA, Padova, Italy) showed foveal fixation in the LE, whereas fixation in the RE was non-foveal. Both eyes demonstrated an abnormal macular integrity index (100%) and reduced mean retinal sensitivity, with average thresholds of 13.4 dB in the RE and 3.0 dB in the LE. Fixation stability was reduced in both eyes, with unstable fixation patterns (RE, P1 = 12% and P2 = 41%; LE, P1 = 18% and P2 = 45%).

**Figure 2:**
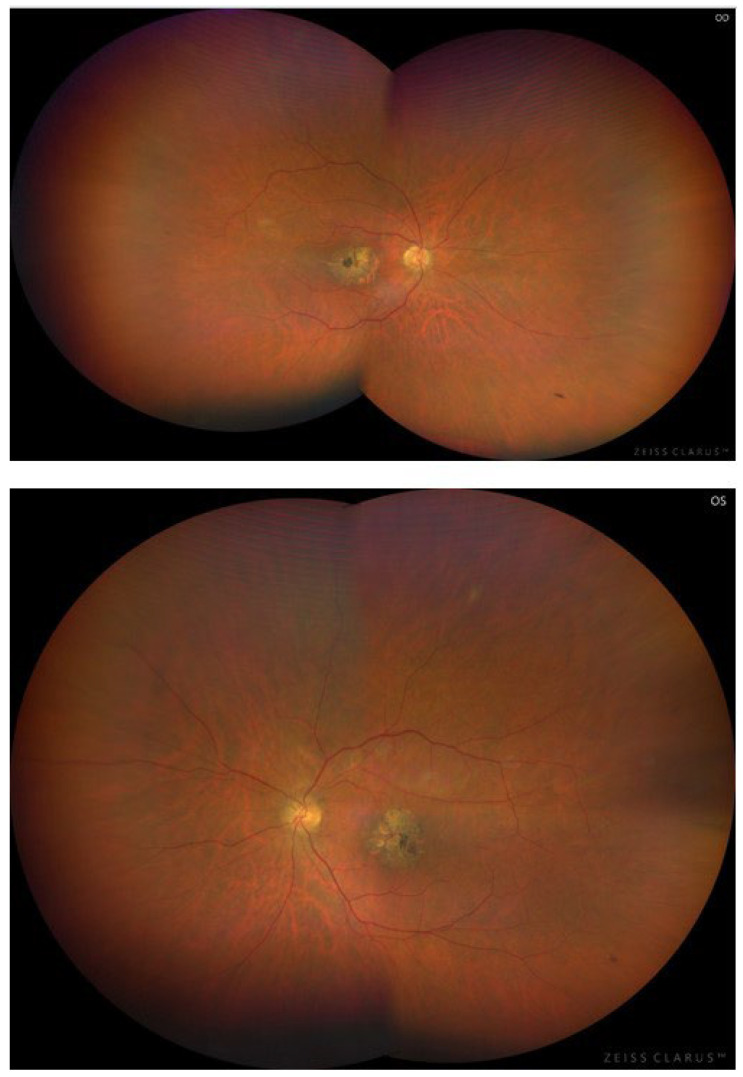
Color fundus photography of both eyes showing bilateral macular atrophy with minimal foveal sparing, accompanied by focal areas of hyperpigmentation, and retinal pigment epithelium (RPE) atrophy. Retinal vessel caliber is preserved, with no evidence of vascular attenuation or arteriolar sclerosis. The optic nerve heads appear slightly tilted, with some peripapillary atrophy while maintaining normal pink neuroretinal rim coloration. No peripheral pigmentary changes are visible.

**Figure 3:**
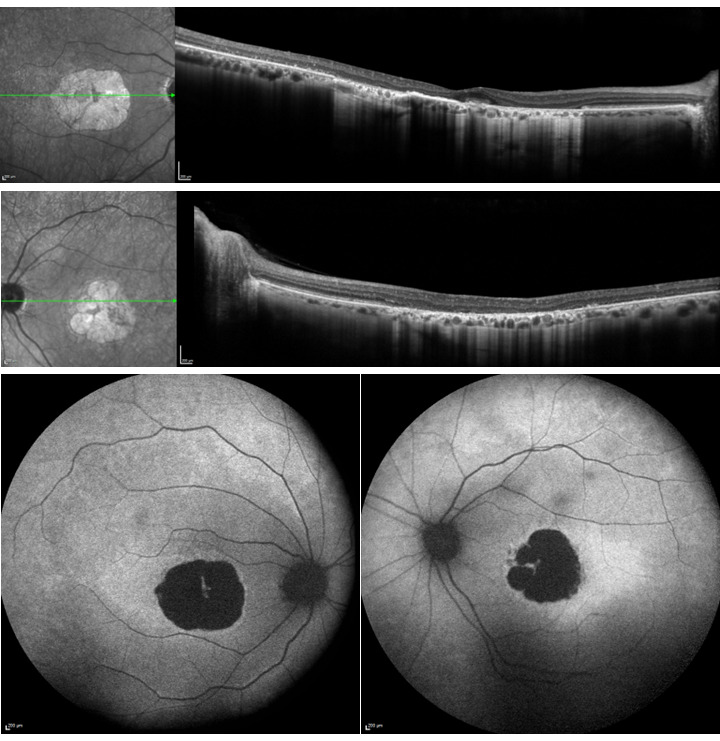
Infrared image and spectral-domain OCT of both eyes showing macular atrophy, with thinning of outer retinal layers and complete loss of the ellipsoid zone (EZ). Areas of RPE atrophy show increased choroidal hypertransmission. Hyperreflective deposits are present in regions, with partial preservation of the outer retina. A reduction in choriocapillaris thickness is also evident. FAF imaging shows a well-demarcated round area of DDAF with minimal foveal sparing, surrounded by a hyperautofluorescent ring. The DDFA lesion measured approximately two disks in size. Additional subtle mottled hyperautofluorescent changes are visible in the superior retina and temporal to the atrophic lesion. No other hypoautofluorescent changes are identified.

Fundus examination demonstrated bilateral macular atrophy with minimal central sparing, along with focal areas of hyperpigmentation and retinal pigment epithelium (RPE) atrophy (Figure 2). No peripheral pigmentary changes were observed, and the optic nerve (ON) head and retinal vessel calibers appeared normal. OCT revealed macular atrophy with disruption of the outer retinal layers, including discontinuity of the external limiting membrane (ELM), loss of the ellipsoid zone (EZ), and RPE alterations. In areas with more advanced RPE damage, a reduction in choriocapillaris (CC) thickness was observed. Infrared imaging showed relatively preserved regions that nevertheless exhibited subtle structural abnormalities on OCT. Peripapillary retinal nerve fiber layer (pRNFL) thickness was preserved. FAF imaging demonstrated central definitive decreased hypo autofluorescence (DDFA) with minimal foveal sparing on short wavelength FAF, surrounded by a hyperautofluorescent ring with temporal extension. Some faint mottled hyperautofluorescent changes could be seen outside the macular area (Figure 3).

### 3.2. Genetic Findings

Genetic analysis identified a homozygous mutation in the *PCARE* gene (NMIM 613425), c.3289C>T, p.Gln1097X), resulting in a premature stop codon.

The variant c.3289C>T (p.Gln1097X) (NM_001029883.2) is classified as pathogenic according to the American College of Medical Genetics and Genomics (ACMG) criteria based on the following evidence:

- The variant is a nonsense change predicted to result in a truncated protein or nonsense-mediated decay. The loss of function is a well-established disease mechanism for *PCARE* (PVS1_very strong).
- The variant has presented at a very low frequency in population databases (GnomAD frequency 0.0026%) (PM2_supporting).
- This variant has been detected in a homozygous or compound heterozygous state with another pathogenic variant in affected individuals, with a recessive disease phenotype (PMID:26306921) (PM3_moderate).
- This variant has been classified as pathogenic in reputable databases. (PP5_supporting).

### 3.3. Electrophysiological Assessment

**Figure 4:**
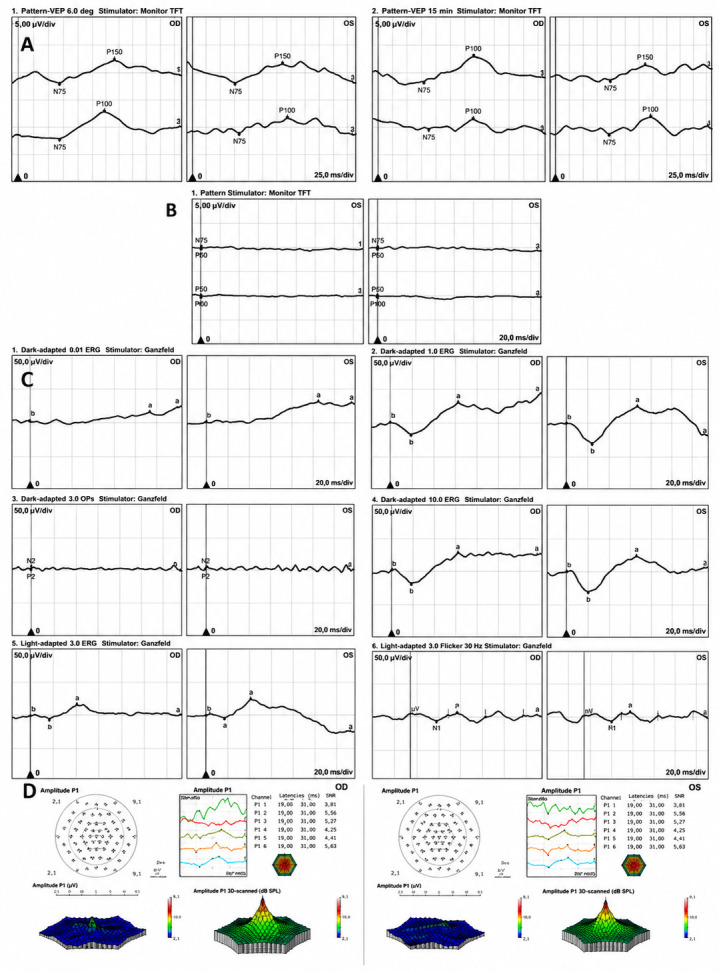
Electrophysiological findings. (**A**) Pattern visual evoked potentials (pVEPs) showing prolonged latencies with reduced amplitudes. (**B**) Pattern electroretinogram (pERG) showing absent or non-reproducible responses. (**C**) Full-field electroretinogram (ffERG; DA 0.01, DA 3.0, DA OPs 3.0, DA 10.0, LA 3.0, and LA 30 Hz flicker) revealing a bilateral reduction in both rod and cone responses, with absent oscillatory potentials (OPs). (**D**) Multifocal electroretinogram (mfERG) demonstrating a severe bilateral reduction in response density across all rings within the central 30° field. Abbreviations: pVEP, pattern visual evoked potential; pERG, pattern electroretinogram; ffERG, full-field electroretinogram; mfERG, multifocal electroretinogram; DA, dark-adapted; LA, light-adapted; OPs, oscillatory potentials.

Pattern visual evoked potentials (pVEPs) showed bilaterally prolonged latencies, with reduced amplitudes and disorganized waveform morphology (Figure 4A).

Pattern electroretinography (pERG) revealed absent or non-reproducible responses in both eyes (Figure 4B).

Full-field electroretinography (ffERG) demonstrated a generalized bilateral reduction in amplitudes affecting both rod- and cone-mediated responses. The scotopic DA 0.01 response was either extinguished or markedly reduced. Mixed rod–cone responses (DA 3.0 and DA 10.0) showed significantly reduced a- and b-wave amplitudes, without evidence of an electronegative pattern. Oscillatory potentials (DA OPs) were absent. Photopic responses (LA 3.0 and 30 Hz flicker) were also markedly reduced (Figure 4C). The a-wave and b-wave values for DA 10.0 amplitudes and implicit times were RE a-wave 18.94 µV, 18.5 ms; RE b-wave and 46.01 µV, 61.5 ms; LE a-wave 29.83 µV, 19.6 ms; and LE b-wave 54.17 µV, 64.7 ms. For LA 3.0 amplitudes and implicit times, these were RE a-wave 4.59 µV, 17 ms; RE b-wave 21.04 µV, 41.6 ms; LE a-wave 3.71 µV, 17 ms; and LE b-wave 29.01 µV, 41.3 ms. Flicker a-wave amplitudes were 11.89 and 12.54 µV for RE and LE, respectively.

Multifocal electroretinography (mfERG) revealed a severe bilateral reduction in response density across all rings within the central 30° field (Figure 4D).

Overall, the electrophysiological findings are consistent with severe diffuse bilateral retinal dysfunction, predominantly affecting rod-mediated pathways with associated cone involvement. No electronegative pattern was identified. Additionally, there was evidence of severe diffuse macular dysfunction, as demonstrated by mfERG.

## 4. Discussion

Null variants are the most frequently reported mutations in IRDs associated with *PCARE*. To date, 87 disease-causing missense or nonsense variants have been documented in the Human Gene Mutation Database (HGMD Professional 2024). In the present study, we describe a patient with CORD who was initially misdiagnosed with Stargardt disease type 1 because of the characteristic DDAF pattern, although no flecks were seen. Although Stargardt disease is by far the most common inherited macular dystrophy, distinguishing it from the early stages of CORD may be challenging [12]. In our patient, electrophysiological testing clearly demonstrated bilateral dysfunction of both cone- and rod-mediated pathways. It is important to recognize that *ABCA4*-associated retinopathy can also present with combined cone and rod dysfunction. Patients classified as group 3 in the electrophysiological staging system for Stargardt disease exhibit severe generalized abnormalities in both photopic and scotopic ffERG responses, thereby mimicking a primary CORD phenotype [13]. *PCARE* mutations represent a rare but important differential diagnosis for this specific phenotype.

Frameshift and nonsense variants that introduce a premature stop codon, resulting in a predicted loss-of-function effect, are the most common mutations identified in *PCARE* [6,14]. Most reported variants are single nucleotide variant or indels [1]. The critical role of the *PCARE* gene in photoreceptor function likely explains the severe forms of RP and the early involvement of cone photoreceptors observed in affected individuals. The *PCARE* protein regulates the initial formation of outer segment disks, working as an actin modulator [1,15]. Consequently, *PCARE*-associated IRDs are considered ciliopathies.

The clinical variability of *PCARE*-associated dystrophies is well-documented, with considerable differences in phenotypic expression and prevalence among geographic regions and study cohorts. RP is the most frequently reported clinical phenotype; however, electrophysiological testing often reveals severe photoreceptor degeneration with early cone involvement [16]. In contrast, several studies have highlighted a clear CORD spectrum associated with *PCARE* mutations. Lopez-Rodriguez et al. [6] reported one of the largest cohorts to date, comprising 14 Latin American patients with CORD caused by biallelic *PCARE* mutations. In their study, the diagnosis of CORD was supported by greater functional preservation under scotopic rather than photopic conditions on chromatic perimetry. Compared with our patient, affected individuals exhibited an earlier disease onset, ranging from the first to the fourth decade of life, with nyctalopia frequently reported as the initial symptom. Most patients showed peripheral bone-spicule pigmentation or hypopigmented deposits, along with optic disk pallor and vascular attenuation. Genetic analysis identified six different *PCARE* variants, three of which were novel and five were null variants. Similarly, in a Swiss cohort, four *PCARE* variants were associated with RP and one with CORD, all of which were truncating mutations [7]. Conversely, a Spanish study of 1036 families with autosomal recessive or sporadic macular dystrophies screened for more than 200 genes did not identify any *PCARE* variants, reinforcing the predominance of *ABCA4* mutations in that population [17]. In France, *PCARE* mutations were identified in approximately 1% of autosomal recessive RP cases and were frequently associated with macular atrophy [16]. In a cohort of 13 patients with *PCARE*-associated retinopathy, early maculopathy was a prominent feature, while fewer than half of the patients complained of nyctalopia. Visual decline typically began during the second to third decade of life [18].

Bianco et al. [14] described natural history of *PCARE*-associated retinopathy in a cohort of 28 patients during a 7-year follow-up period. All individuals exhibited severe photoreceptor degeneration accompanied by macular atrophy. The median age at first examination was 40.7 years, closely matching that of our patient. DDAF within the macula was present in approximately 85% of cases, while foveal sparing was common at diagnosis, with a median age of progression to foveal atrophy of 45.3 years. However, FAF abnormalities frequently extended beyond the macular region. The mean rate of DDAF enlargement was estimated at 0.20 mm/year, comparable to that reported in *ABCA4* associated retinopathies. Genetic analysis identified 25 different variants, including frameshift (52%), nonsense (40%), and missense (8%) variants. Similarly, in the 13-patient cohort studied by Gerth-Kahlert et al., where the mean age was 32 years, younger patients exhibited more severe cone involvement, suggesting that cone involvement may be an early and prominent feature of the disease [18]. None of the patients in the two largest reported *PCARE* cohorts carried the variant identified in our patient [14,18]. However, Khateb et al. described the same *PCARE* mutation (c.3289C>T) in combination with a homozygous *CEP250* variant, c.3463C>T (p.Arg1155*). *CEP250* encodes the centrosome-associated protein CEP250 (also known as C-Nap1), and the coexistence of pathogenic variants in both genes was associated with an atypical form of Usher syndrome [19]. The presence of nonsense variants in two genes involved in ciliary function raises the possibility of an additive or synergistic effect contributing to disease severity. Consistent with this hypothesis, individuals carrying a homozygous *CEP250* mutation, together with a heterozygous *C2orf71* variant, exhibited a relatively mild retinal phenotype, whereas patients who were homozygous for pathogenic variants in both genes showed more severe retinal involvement. These observations support the concept of genetic interaction between ciliary disease genes as a potential modifier of phenotypic expression.

Further evidence of the long-term progression of *PCARE*-associated disease was provided by Serra et al., who reported a 45-year-old Italian man with early-onset CORD caused by a *PCARE* mutation [5]. The patient initially presented at 25 years of age with subacute bilateral central vision loss. Fundus examination revealed perifoveal atrophy and optic disk pallor without pigment deposition. ERG demonstrated reduced rod and cone amplitudes, with greater impairment under photopic conditions, consistent with CORD. Over a 20-year follow-up period, VA deteriorated to light perception in both eyes, accompanied by complete visual field loss. FAF imaging revealed central macular atrophy with patchy mid-peripheral involvement, while spectral-domain OCT demonstrated marked bilateral retinal thinning with loss of the outer retinal layers [5]. Several reports have described FAF findings similar to those observed in our patient, although these are typically accompanied by hypoautofluorescent changes in the mid peripheral retina [6,14]. Bianco et al. reported a 22-year-old patient carrying the homozygous *PCARE* variant c.1541del [p.(Pro514Hisfs*27)]. During a 12-year follow-up, the patient developed a central DDAF area without foveal sparing on OCT. In addition, mottled hypoautofluorescent lesions were observed along the retinal vessels, together with loss of the outer retina hyperreflective band beyond the macular region.

The age at onset of *PCARE* retinopathy varies widely, from the first to the sixth decade of life. Nyctalopia is reported in less than 50% of affected subjects, whereas reduced VA is the most common presenting symptom. Visual impairment is generally associated with macular atrophy, and ffERG typically demonstrates early dysfunction of both rod and cone photoreceptors [18]. Patchy RPE atrophy in the mid-peripheral retina is also frequently observed, whereas bone spicule pigmentation is relatively uncommon.

Our patient reported progressive visual loss beginning in the fifth decade of life, without nyctalopia or photophobia. ffERG revealed abnormalities in both rod- and cone-mediated responses, and macular atrophy was the predominant retinal finding. Notably, no additional round atrophic lesions were detected in the mid-peripheral retina. OCT imaging showed preservation of the outer retinal layers outside the central atrophic area, suggesting relative preservation of rod photoreceptors. Although FAF did not reveal the fleck-like lesions typically associated with *ABCA4*-related retinopathy, the presence of a central atrophic lesion with foveal sparing initially suggested a late-onset *ABCA4*-associated phenotype. Progressive central atrophy is one of the hallmark features of *ABCA4* retinopathy. Additional characteristic findings include fundus flecks, which were absent in our patient, and peripapillary sparing, which was present. However, numerous genetic phenocopies of *ABCA4*-related disease have been described, including other genes associated with Stargardt disease, such as *ELOVL4* and *PROM1*, as well as genes causing macular dystrophies with overlapping clinical presentations, including *CRX*, *MT-TL1*, *PRPH2*, *RDH12*, and *RPGR* [20].

Currently, no approved treatment exists for *PCARE*-associated retinal degeneration. Nevertheless, the availability of both rodent and canine disease models, together with the relatively small size of the *PCARE* coding sequence, makes this gene an attractive candidate for adeno-associated virus (AAV)-mediated gene augmentation therapy [2,21].

In conclusion, this case underscores the significant phenotypic overlap between early-stage CORD and Stargardt disease, emphasizing the risk of misdiagnosis when interpretation relies primarily on FAF findings. Although *ABCA4* remains the predominant cause of inherited macular dystrophies, *PCARE* mutations should be considered in atypical presentations, particularly when electrophysiological findings suggest widespread photoreceptor dysfunction. Together with the existing literature, our findings highlight the marked clinical heterogeneity and geographic variability of *PCARE*-associated retinopathies. These observations reinforce the critical role of comprehensive electrophysiological testing and molecular genetic screening in establishing an accurate diagnosis, guiding prognostic counseling, and appropriate management of IRD patients.

## Data Availability

The data presented in this study are available on request from the corresponding author due to legal and ethical reasons.

## Appendix A

NGS Analysis of Clinically Relevant Exonic and Intronic Regions:

ABCA4, ABCC6, ABHD12, ACBD5, ACO2, ADAM9, ADAMTS18, ADGRV1, ADIPOR1, AFG3L2, AGBL5, AHI1, AIPL1, ALMS1, AMACR, AP3B1, AP3D1, ARHGEF18, ARL13B, ARL2BP, ARL3, ARL6, ARSG, ASRGL1, ATF6, ATOH7, ATP1A3, ATXN7, AUH, B9D1, B9D2, BBIP1, BBS1, BBS10, BBS12, BBS2, BBS4, BBS5, BBS7, BBS9, BEST1, BLOC1S3, BLOC1S5, BLOC1S6, C12orf65, C19orf12, C1QTNF5, C8orf37, CABP4, CACNA1F, CACNA2D4, CAPN5, CAV1, CC2D2A, CCT2, CDH23, CDH3, CDHR1, CEP120, CEP164, CEP250, CEP290, CEP78, CEP83, CERKL, CHM, CISD2, CLN3, CLN5, CLN6, CLN8, CLRN1, CNGA1, CNGA3, CNGB1, CNGB3, CNNM4, COL11A1, COL18A1, COL2A1, COL4A1, COL9A1, COL9A2, COL9A3, COQ2, CRB1, CRX, CSPP1, CTC1, CTNNA1, CTNNB1, CTNND1, CTSD, CWC27, CYP4V2, DCT, DHDDS, DHX38, DNAJC30, DRAM2, DTNBP1, DYNC2H1, EFEMP1, ELOVL1, ELOVL4, EMC1, ERCC8, EXOSC2, EYS, FAM161A, FDXR, FLVCR1, FRMD7, FZD4, GNAT1, GNAT2, GNB3, GPR143, GPR179, GRK1, GRM6, GUCA1A, GUCA1B, GUCY2D, HARS, HCCS, HGSNAT, HK1, HMX1, HPS1, HPS3, HPS4, HPS5, HPS6, HTRA1, IDH3A, IDH3B, IFT140, IFT172, IFT27, IFT74, IKBKG, IMPDH1, IMPG1, IMPG2, INPP5E, INVS, IQCB1, JAG1, KCNJ13, KCNV2, KIAA0556, KIAA1549, KIF11, KIF3B, KIF7, KIZ, KLHL7, LAMA1, LCA5, LRAT, LRIT3, LRMDA, LRP2, LRP5, LYST, LZTFL1, MAK, MAPKAPK3, MCOLN1, MED12, MERTK, MFN2, MFRP, MFSD8, MKKS, MKS1, MLPH, MSTO1, MT-ATP6, MT-ATP8, MT-CO1, MT-CO2, MT-CO3, MT-CYB, MT-ND1, MT-ND2, MT-ND3, MT-ND4, MT-ND4L, MT-ND5, MT-ND6, MT-RNR1, MT-RNR2, MT-TA, MT-TC, MT-TD, MT-TE, MT-TF, MT-TG, MT-TH, MT-TI, MT-TK, MT-TL1, MT-TL2, MT-TM, MT-TN, MT-TP, MT-TQ, MT-TR, MT-TS1, MT-TS2, MT-TT, MT-TV, MT-TW, MT-TY, MTPAP, MTTP, MVK, MYO5A, MYO7A, NBAS, NDP, NEUROD1, NMNAT1, NPHP1, NPHP3, NPHP4, NR2E3, NR2F1, NRL, NYX, OAT, OCA2, OFD1, OPA1, OPA3, OPN1LW, OPN1MW, OPN1SW, OTX2, P3H2, PANK2, PAX2, PCARE, PCDH15, PCYT1A, PDE6A, PDE6B, PDE6C, PDE6G, PDE6H, PDSS1, PEX1, PEX10, PEX11B, PEX12, PEX13, PEX14, PEX16, PEX19, PEX2, PEX26, PEX3, PEX5, PEX6, PEX7, PHYH, PLA2G5, PNPLA6, POC1B, POMGNT1, POMT1, PPT1, PRCD, PRDM13, PROM1, PRPF3, PRPF31, PRPF4, PRPF6, PRPF8, PRPH2, PRPS1, PYGM, RAB27A, RAB28, RAX2, RBP3, RBP4, RCBTB1, RD3, RDH11, RDH12, RDH5, REEP6, RGR, RGS9, RHO, RIMS1, RIMS2, RLBP1, ROM1, RP1, RP1L1, RP2, RPE65, RPGR, RPGRIP1, RPGRIP1L, RS1, RTN4IP1, SAG, SAMD11, SAMD7, SCAPER, SDCCAG8, SDHA, SEMA4A, SLC24A1, SLC24A5, SLC25A46, SLC37A3, SLC38A8, SLC45A2, SLC52A2, SLC6A6, SLC7A14, SNRNP200, SPATA7, SPG7, SRD5A3, SSBP1, STN1, TCTN1, TCTN2, TCTN3, TEAD1, TIMM8A, TIMP3, TINF2, TMEM126A, TMEM216, TMEM218, TMEM231, TMEM67, TOPORS, TPP1, TRAF3IP1, TREX1, TRNT1, TRPM1, TSPAN12, TTC21B, TTC8, TTLL5, TTPA, TUB, TUBB4B, TUBGCP4, TUBGCP6, TULP1, TYR, TYRP1, UCHL1, UNC119, USH1C, USH1G, USH2A, USP45, VCAN, VPS13B, WDR19, WFS1, ZFYVE26, ZNF408, ZNF423, ZNF513.
